# A classification model for lncRNA and mRNA based on k-mers and a convolutional neural network

**DOI:** 10.1186/s12859-019-3039-3

**Published:** 2019-09-13

**Authors:** Jianghui Wen, Yeshu Liu, Yu Shi, Haoran Huang, Bing Deng, Xinping Xiao

**Affiliations:** 1 School of Science, Wuhan University of Technology, Wuhan, 430070 People’s Republic of China; 2 Wuhan Academy of Agricultural Sciences, Wuhan, 430208 People’s Republic of China

**Keywords:** lncRNA, mRNA, K-mers, Relative entropy, Convolutional neural network

## Abstract

**Background:**

Long-chain non-coding RNA (lncRNA) is closely related to many biological activities. Since its sequence structure is similar to that of messenger RNA (mRNA), it is difficult to distinguish between the two based only on sequence biometrics. Therefore, it is particularly important to construct a model that can effectively identify lncRNA and mRNA.

**Results:**

First, the difference in the k-mer frequency distribution between lncRNA and mRNA sequences is considered in this paper, and they are transformed into the k-mer frequency matrix. Moreover, k-mers with more species are screened by relative entropy. The classification model of the lncRNA and mRNA sequences is then proposed by inputting the k-mer frequency matrix and training the convolutional neural network. Finally, the optimal k-mer combination of the classification model is determined and compared with other machine learning methods in humans, mice and chickens. The results indicate that the proposed model has the highest classification accuracy. Furthermore, the recognition ability of this model is verified to a single sequence.

**Conclusion:**

We established a classification model for lncRNA and mRNA based on k-mers and the convolutional neural network. The classification accuracy of the model with 1-mers, 2-mers and 3-mers was the highest, with an accuracy of 0.9872 in humans, 0.8797 in mice and 0.9963 in chickens, which is better than those of the random forest, logistic regression, decision tree and support vector machine.

## Background

Transcription of the genome includes messenger RNAs (mRNAs), small (miRNAs, snRNAs) and non-coding RNAs (ncRNAs) [1, 2]. LncRNA is a kind of noncoding RNA with a length exceeding 200 nucleotides [3]. There is a growing concern over the long non-coding RNA [4]. Current studies demonstrate that lncRNA sequences primarily play a role in two aspects of the organism. On one hand, they play a vital biological function in many stages, such as transcription and regulation of life processes. For example, lncRNAs can participate in the regulation of gene expression levels at three levels, epigenetic regulation, transcriptional regulation and post-transcriptional regulation [5], and some lncRNAs can bind to specific chromatin-related sites through chromatin remodelling, resulting in the expression of silenced related genes [6, 7]. On the other hand, lncRNAs have direct or indirect links with some diseases in humans, such as lung cancer, prostate cancer, Alzheimer’s disease, Prader-Willi syndrome, agitation, etc. [4]. Therefore, the identification and inclusion of lncRNAs will help researchers to further research and explore their functions at the molecular level [8].

However, thus far, only a small number of lncRNAs have been included in the non-coding RNA-related database. Additionally, in the existing database, only a small number of lncRNA functions have been thoroughly studied and annotated. Even more difficult is that functional studies of the lncRNA sequence are based on the premise that they can determine whether the sequence is a lncRNA, which is the main difficulty in biological and information biology research. Since lncRNAs and mRNAs have many similarities in sequence structure, the task of identifying lncRNA sequences becomes more challenging. Accordingly, how to design a model that can accurately identify lncRNA and mRNA sequences based on the large amount of sequence data obtained by high-throughput sequencing will be an important biological research topic.

At present, research mainly classifies coding RNA and non-coding RNA based on three aspects: first is the discrimination by the length of the open reading frame of the coding sequence and the non-coding sequence, second is the discrimination by comparing the similarity between the sequence and the known protein sequence using the comparative genomics methods, and third is the prediction by conservation of the RNA secondary structure. However, each of these three methods has its own merits and demerits, and it is difficult to acquire accurate sequence classification results based on only one of them. To solve this problem, some scholars have constructed models and software for classifying mRNAs and lncRNAs by extracting non-coding features in lncRNA sequences. For example, the Bioinformatics Center of Peking University had developed an online lncRNA identification tool, CPC (Coding Potential Calculator, CPC) [9], which has been widely used in many fields, such as sequence alignment, disease research and evolution analysis. Its principle is mainly to extract six features, containing the ratio of the length of the open reading frame to the sequence length, the integrity of the open reading frame, the prediction reliability evaluation score of the open reading frame, etc., train data by placing those features into a support vector machine (SVM), and develop a prediction model of non-coding RNA. Sun et al. [10] proposed a method named CNCI (Coding-Non-Coding Index, CNCI) based on Adjoining Nucleotide Triplets (ANT). Its framework consisted of a scoring matrix and classification model. First, the species categories of the sample data were determined, and the probability of occurrence of each pair of adjacent triplets in the coding region, non-coding region and inter-gene region was respectively counted to construct three ANT probability matrices. Then, as the reference, the log-ratio of the ANT probability matrix of the coding and non-coding region were respectively calculated to obtain the scoring matrix of the CNCI algorithm. Further, The CNCI scoring matrix was used to determine the Most-Like Coding Domain Sequence (MLCDS), and then five different features were extracted from each MLCDS for the classification. Dang [11] selected three characteristics from the perspective of the open reading frame, three characteristics of the integrated sequence secondary structure and two characteristics of protein similarity and summarized seven combinations of three types of features. Her lncRNA prediction model could be suitable for different data source.

The CSF prediction software proposed by Lin et al. [12] mainly aimed to identify lncRNAs by calculating the frequency of codon substitution in the target sequence. Based on the CSF model, the evolution information of the alignment sequence was introduced, and they developed the Phylo CSF recognition model [13]. Wucher et al. developed the FEELnc program, an lncRNA and mRNA recognition tool, which was a random forest-based classification model trained using features such as open reading frames [2]. In 2014, Lertampaiporn et al. developed a hybrid model based on logistic regression and random forests to distinguish short non-coding RNA sequences from lncRNA sequences. The model synthesized five combined features, SCORE, which improved the lncRNA classification performance [14].

To summarize, most of the available methods for identifying lncRNA among mRNA sequences are based on the biological characteristics of the sequences. However, the lncRNA sequence may contain some sequences that can overlap with the coding regions of mRNAs [2]. Thus, the recognition of lncRNA sequences is more complex than the recognition of mRNA sequences when using existing methods. To avoid the use of sequence biological characteristics to establish a classification model of the sequence, Wei proposed an lncRNA and mRNA classification model based on the k-mer [15]. This model used the maximum entropy algorithm to screen k-mers and the support vector machine algorithm for classification; however, it demonstrated great computational complexity and a high computational cost. In addition, pre-processing of raw input data and sequence features should be selected by domain-expert knowledge and to fine-tune parameters to increase accuracy when using conventional machine learning algorithms such as support vector machines, logistic regression, decision trees, SVM, NN, BNs, GAs, and HMMs, etc. [16]. Therefore, we propose a model to effectively classify lncRNAs and mRNAs, without relying on the sequencing quality and biological structural characteristics of the sequence, as well as avoiding a large number of calculations.

Since the Convolutional Neural Network (CNN) model can self-learn the characteristics of the sequence through continuous training without artificial intervention and efficiently calculate large amounts of data, no domain-expert knowledge or fine-tuning of parameters to increase accuracy are needed [17, 18]. It has been used to predict DNA-protein binding sites [19] and to predict the specificity of DNA and RNA binding proteins [20]. Zhang et al., developed two methods for predicting DNA-protein binding using the High-Order Convolutional Neural Network Architecture and Weakly Supervised Convolutional Neural Network Architecture [21, 22]. Transcription factor prediction using ChIP-seq data [23] and CRISPR guide RNA design [24] can also be finely conducted using CNN. Whether CNN can be finely used in the classification of lncRNAs and mRNAs is not known.

In this study, we intend to introduce the convolutional neural network model to establish a classification model of lncRNAs and mRNAs. The content of this paper is arranged as follows. First, the k-mer frequency information for lncRNA and mRNA sequences is statistically analysed. Second, we construct the classification model of lncRNA and mRNA sequences by convolutional neural network taking the k-mer frequency matrix as input. Third, we determine the optimal k-mer combination of the model, compare it with those of other machine learning methods and verify the recognition ability of identifying a single sequence.

## Results and discussion

### Training data and testing data

We download human lncRNA sequence data and mRNA sequence data from the GENCODE database, gencode.v 26. The 10,000 sequences data are randomly selected from the two sample sets each time, i.e., 10,000 lncRNA sequences and 10,000 mRNA sequences, of which 8000 sequences are selected as training samples and the remaining 2000 sequences are used as test samples. We perform 10 random selections to verify the contingency impact of the randomly selected data training model.

**Fig. 1:**
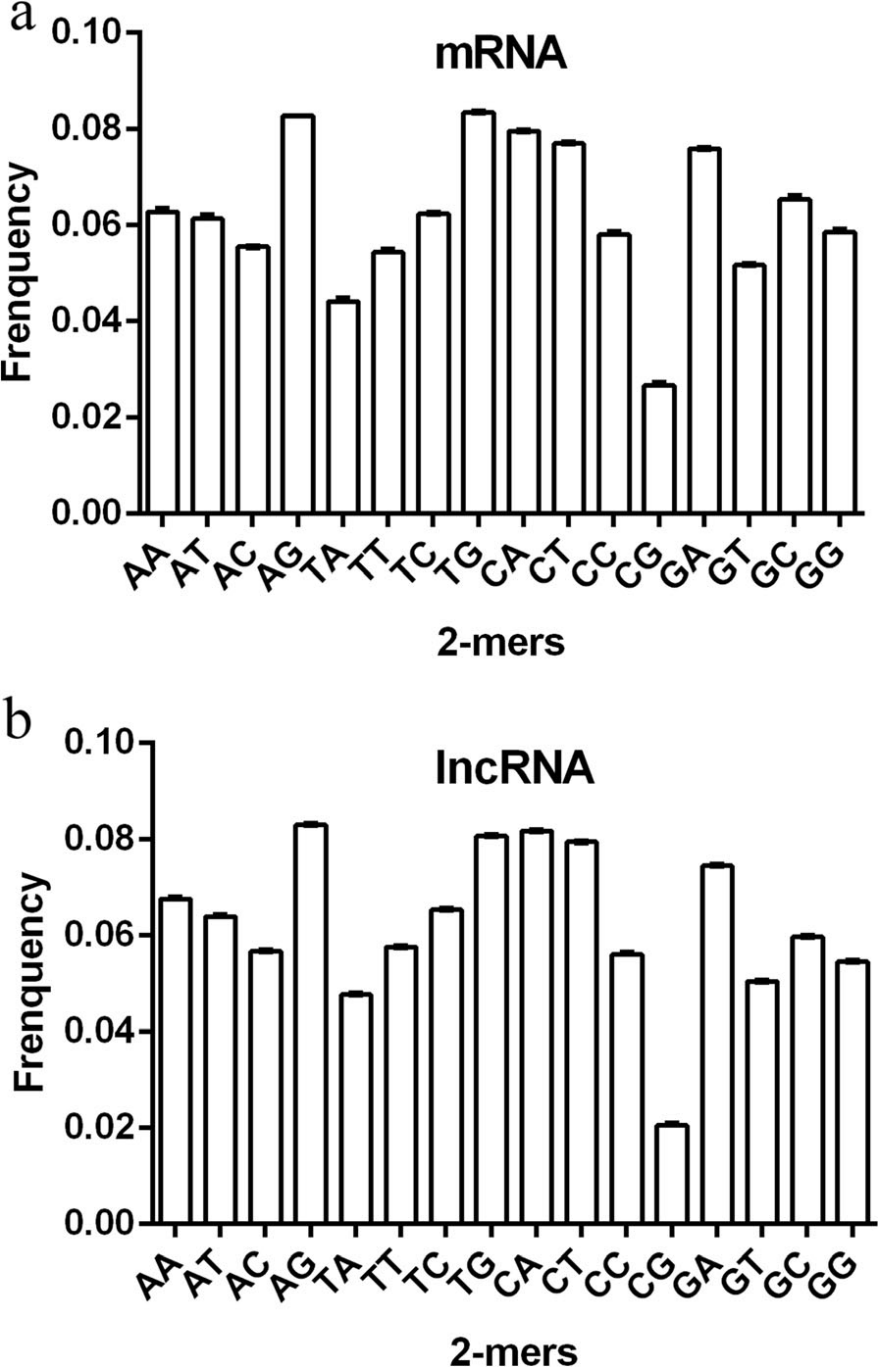
The 2-mer frequency mean line graph. **a** The 2-mer frequency mean line graph of lncRNA. **b** The 2-mer frequency mean line graph of mRNA

The frequency means of 2-mers in the 10 sets of sequences are calculated, and the line graphs are shown in Fig. 1a and b. In Fig. 1, the lncRNA mean line graphs in the 10 sets of data almost coincide. Only the AA of the first set of data is slightly different from the other groups. The average AA frequency of the first set of data is 0.069, the average AA frequency of the second, third, fourth, sixth, eighth, ninth, and tenth sets of data is 0.067, while the fifth set is 0.068. It can be seen that the difference between the data does not exceed 0.002, and the error is small. From Fig. 1b, the mRNA means line graphs in the 10 sets of data also show mostly overlap, and only the four k-mers of AA, AT, GC, and GG differ. However, the data show that the extremes of the frequency means of AA, AT, GC, and GG in the 10 sets of data are approximately 0.0048, 0.0048, 0.0046, and 0.0036 respectively. Thus, the differences between are not large. Therefore, the randomness of the data extraction does not greatly affect the calculation results of the model.

### Determination of k-mer parameters of the lncRNA classification model

First, the calculation is divided into two steps. In the first step, lncRNA sequences ranging from 250 nt to 3500 nt and mRNA sequences ranging from 200 nt to 4000 nt are selected. The k-mer subsequence is extracted using the k-mer algorithm. For the k-mers with larger values, the relative entropy is used to select the features, and the accuracy of the model before and after the screening is compared. Finally, the frequency of the k-mer subsequence in each sequence is counted, and the frequency matrix is constructed. In the second step, the convolutional neural network model is trained using the constructed frequency matrix to obtain the classification results of the model. When *k* is taken different values, the results are compared to obtain the optimal classification model parameters.

### Determination of optimal k-mer combination in the lncRNA classification model

Based on the statistical analysis, we randomly select 10,000 lncRNA sequence data ranging from 250 nt to 3000 nt in the lncRNA dataset downloaded from the GENCODE database, and we also randomly select 10,000 mRNA sequence data ranging from 200 nt to 4000 nt in length.

Next, we build an lncRNA and mRNA classification model. The first layer of the convolutional neural network uses 32 convolution kernels of 3 × 3, selects the Relu activation function, and the periphery of the k-mer frequency matrix is padded with “0” to ensure a constant size of the matrix before and after the convolution calculation. The second layer is still the convolutional layer, with 64 convolution kernels of 3 × 3, and the activation function is still the Relu function. The third layer is the largest pooling layer, and the size of the pooling area is 2 × 2. The partial neuron connections with a probability of 0.25 are omitted before the fully connected layer to prevent overfitting. The last layer is the fully connected layer. There are 128 neurons in the fully connected layer. After the whole layer is connected, the probability of connections between the omitted neurons is 0.5. Finally, the SoftMax function is used to obtain the classification result. The loss function in the model training process selects the cross entropy loss function, and the optimizer is Adadelta.

To determine the most differential k-mers in lncRNA and mRNA sequences and to maximize the accuracy of the model k-mers, we select k-mers with different *k* values. The established lncRNA and mRNA classification models are used to learn autonomously. Finally, the classification accuracy, model accuracy, recall rate and *F*_1_ score of the classification model are compared when different *k* values are compared.

We take a single *k* value, which is 3, 4, 5, and 6. The specific results are shown in Table 1.

**Table 1:** Model classification accuracy for individual *k* value

*k*value	number of k-mers	matrix form	model accuracy	precision rate(P)	recall rate(R)	*F*_1_score	calculating time (s/epoch)
3	64	8 × 8	0.7508	0.81	0.79	0.79	5
4	256	16 × 16	0.7610	0.85	0.83	0.83	20
5	1024	32 × 32	0.7565	0.93	0.92	0.92	95
6	4096	64 × 64	0.7748	0.87	0.85	0.84	855

It can be seen from Table 1 that the classification effect of the model is different when different *k* values are taken. As the *k* value increases, the number of k-mers increases and the model accuracy generally increases. However, when *k* = 5, then the accuracy of the classification model is slightly lower than *k* = 4, but the difference does not exceed 0.01, and the difference is not large. When there are too many types of k-mers, the frequency of each k-mer will also decrease, and even the frequency of most k-mers will be 0, so each k-mer will carry less difference information. When *k* is 6, the accuracy is the highest, but it is only 0.7748. This classification is not ideal, and its time complexity is the highest because it requires 855 s to calculate a time of 1 epoch. However, when *k* is equal to 3, the accuracy is only approximately 0.024 lower than it is with *k* = 6, but it takes only 5 s to calculate 1 epoch. Therefore, we attempt to combine these individual k-mers in pairs and analyse the results to determine whether this attempt is reasonable. The specific calculation results are shown in Table 2.

**Table 2:** Model classification accuracy rate of two *k* value combinations

*k*value	number of k-mers	matrix form	model accuracy	precision rate(P)	recall rate(R)	*F*_1_score	calculating time (s/epoch)
1 + 3	68	17 × 4	0.9280	0.94	0.94	0.94	4
1 + 4	260	10 × 26	0.9600	0.98	0.98	0.98	32
1 + 5	1028	4 × 257	0.4995	0.50	0.50	0.36	43
2 + 3	80	8 × 10	0.9810	0.99	0.99	0.99	9
2 + 4	272	16 × 17	0.7838	0.87	0.86	0.86	37
2 + 5	1040	26 × 40	0.7672	0.91	0.90	0.90	180
3 + 4	320	16 × 20	0.7666	0.90	0.90	0.90	47
3 + 5	1088	32 × 34	0.7566	0.94	0.94	0.94	189
4 + 5	1280	32 × 40	0.7532	0.95	0.94	0.94	290

Since the time complexity is too high when *k* = 6, if the combination calculation is performed, then the calculation time will be unsatisfactory, only taking *k* to be 1, 2, 3, 4, and 5 in pairs. From Table 2, the recognition accuracy of the classification model is significantly improved when we combine the two k-mers, especially the combination of *k* = 2 and *k* = 3, with an accuracy reaching 0.9810. The second is a combination of *k* = 1 and *k* = 4 with an accuracy of 0.9600. The result can be explained by the combination of k-mers, which is equivalent to strengthening the k-mer information of the sequence, after which the model can receive more difference information through convolutional neural network self-learning. However, Table 2 also reveals such information. Although the combined information can greatly improve the accuracy of model recognition, not every combination of information can improve the recognition accuracy of the model compared with before the combination. For example, when *k* = 5, as shown in Table 1, the classification accuracy of the model is 0.7565, and when *k* = 1 and *k* = 5 are combined, the accuracy is 0.4995, and the accuracy of the model is not increased but decreased.

Although the combined k-mers can greatly improve the accuracy of the classification model, this strategy also consumes more computation time than the model of a single k-mer. By comparison, the calculation time of the classification model is proportional to the number of k-mers. When the number of k-mers is larger, the calculation time consumed by the model is also greater. There are 80 k-mers in the combination of *k* = 2 and *k* = 3, and the calculation time consumed by 1 epoch is 9 s, which is second only to the combination of *k* = 1 and *k* = 3. The longest calculation time is the combination of *k* = 4 and *k* = 5, which contains a total of 1280 k-mers. The calculation of 1 epoch requires 290 s. If 200 iterations are obtained, it will take approximately 16 h to train the model. It should be noted that the calculation time in the combination of *k* = 1 and *k* = 3 is 4 s, which is faster than the calculation time in *k* = 3 (5 s). This phenomenon is due to the presence of 68 k-mers in the combination of *k* = 1 and *k* = 3, which is input as 4 × 17 matrix, and 2 × 2 convolution layer for feature extraction. When the number of 3-mers is 64, which is input as 8 × 8 matrix, and, the convolution layer is 3 × 3 for feature extraction. Then the calculation time for the combination of *k* = 1 and *k* = 3 is faster than the calculation time for *k* = 3.

Based on the information presented in Table 2, the combination of *k* = 2 and *k* = 3 provides the classification model with the highest combination information. Furthermore, the computational time cost of this combination is relatively low. Therefore, we attempt to combine *k* = 2 and *k* = 3 with other k-mers and use more combination information to verify whether the combination of k-mers of the three *k* values will further improve the accuracy of the model based on the combination of the two *k* values of k-mers. The specific calculation results are shown in Table 3.

**Table 3:** Model classification accuracy rate of three *k* value combinations

*k*value	number of k-mers	matrix form	model accuracy	precision rate	recall rate	*F*_1_score	calculating time (s/epoch)
1 + 2 + 3	84	17 × 20	0.9872	1.00	1.00	1.00	6
2 + 3 + 4	336	12 × 28	0.9738	1.00	1.00	1.00	57
2 + 3 + 5	1104	24 × 46	0.9798	1.00	1.00	1.00	217

Based on Table 3, we find that the combination of *k* = 1, *k* = 2, and *k* = 3 can further improve the accuracy of the model due to the combination of k-mers, and the recognition accuracy of the model can reach 0.9872, as shown in Table 3. More excitingly, the calculation time is only 6 s, which is far less than that of other k-mer combinations. Consequently, the k-mer combination of *k* = 1, 2, 3 not only achieves the best model accuracy but also has an accuracy rate and recall rate and *F*_1_ score of 1.00, which indicates that the classification effect of the model is also excellent. Based on the above results, we determine the k-mers that allow optimal construction of the lncRNA and mRNA classification model, which is the combination of 1-mers, 2-mers and 3-mers.

### Determination of the optimal combination of k-mers for the selected lncRNA classification model

In the calculation process, we find that when the *k* value is greater than 4, that is, when it is 5 or 6 or more, a considerable portion of the k-mers exhibits a frequency of 0. The lack of most k-mer values may affect the recognition accuracy of the model, resulting in a low classification accuracy of the model. To verify this conjecture, we use the relative entropy to filter the k-mers of *k* = 5 and *k* = 6. By sorting the information gains and selecting the top 98% k-mers, the k-mers carrying more and less difference information are filtered out. This method can also effectively reduce the dimensions of the k-mer frequency matrix.

As shown in Table 4, the k-mers of *k* = 5 are reduced from the original 1024 k-mers to 115 after screening with relative entropy. The 115 k-mers are constructed with a matrix of 5 × 23. Finally, after screening with relative entropy, the 5-mers improve the model accuracy of *k* = 5 from 0.7565 to 0.7820. Similarly, the 6-mers screened by relative entropy show a reduction from the original 4096 to 1045. Although the accuracy of the 6-mers model is slightly improved from 0.7748 to 0.7790, this improvement is almost negligible compared with before the relative entropy screening. Since the number of k-mers reaches as high as 4096 in *k* = 6, the difference information for the sequence becomes very fragmented. Although the relative entropy screening retains 98% of the difference information, the latter part of the information is abandoned. This may explain why the accuracy of the model does not increase significantly.

**Table 4:** K-mers calculation results after KL screening

*k*value	number of k-mers	number of k-mers after KL screening	original model accuracy	model accuracy after KL screening	calculation time of the original model (s/epoch)	calculation time of KL screening model (s/epoch)
5	1024	115	0.7565	0.782	95 s	4 s
6	4096	1045	0.7748	0.779	855 s	47 s
4 + 5	1280	112	0.7532	0.629	290 s	4 s
2 + 3 + 5	1104	195	0.9798	0.9761	217 s	27 s

To compare the classification accuracy of the k-mer combination, we combine the 5-mers after the relative entropy screening with the k-mers of *k* = 4. It is found that the combined k-mer accuracy is only 0.6290, and the accuracy of the model is not improved but reduced. In addition, we combine the 5-mers after the relative entropy screening with the k-mers of *k* = 2 and *k* = 3, and we find that the accuracy of the model is improved to 0.9761, but it is still not the k-mer combination that provides the best model classification.

Although the accuracy of the model is not very obvious after the relative entropy screening, according to the information in Table 4, since the k-mers are screened by relative entropy screening, the number of k-mers is reduced. The computation time of the model after relative entropy screening is greatly reduced. In particular, the combination of *k* = 4 and *k* = 5 reduces the calculation time from 290 to 4 s, a reduction of more than 70 times.

### Comparison of the model accuracy with four machine learning methods

Based on previous analysis, when k-mers of *k* = 1, *k* = 2 and *k* = 3 are combined as input in the convolutional neural network, the accuracy of the classification model can be maximized. In the case of the 10-fold cross-validation calculation, the training set loss function value of the convolutional neural network model averages 0.043, and the average classification accuracy rate is 0.9872. The average loss function of the verification set is 0.0431, and the average accuracy of the verification set is 0.9790. In the machine learning algorithm, such as the random forest, logistic regression, decision tree, and support vector machine, to compare the superiority of the convolutional neural network model in the classification of lncRNA sequences and mRNA sequences, we use these four algorithms to classify lncRNA and mRNA sequences.

For these four machine learning algorithms, we use the same training data set and verification data set as the convolutional neural network model to train and verify the model separately, and we compare the results with the convolutional neural network model. The results are shown in Table 5.

**Table 5:** Five model effect comparison table in human

model	model accuracy	precision rate(P)	recall rate(R)	*F*_1_score
CNN	0.9872	0.9993	0.9955	0.9974
RF	0.8820	0.8949	0.8867	0.8925
LR	0.7020	0.7247	0.7183	0.7218
DT	0.8030	0.7873	0.7852	0.7869
SVM	0.7020	0.7245	0.7158	0.7179

From Table 5, in terms of model accuracy, the model accuracy of the convolutional neural network algorithm is 0.9872, which is far superior to those of other algorithms. Followed by random forests, the classification accuracy is 0.8820. Again, the decision tree has a classification accuracy of 0.8030. The classification accuracy of the logistic regression and support vector machine is the same at only 0.7020.

**Fig. 2:**
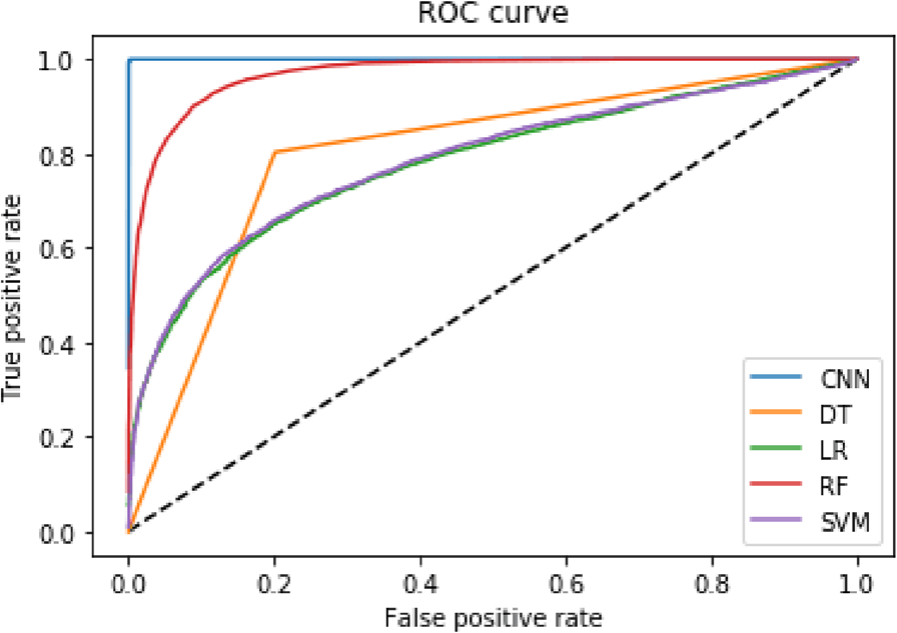
ROC curve of CNN, RF, LR, DT and SVM

The precision rate (P), recall rate (R) and F1 score are also shown in Table 5, all of which are superior to RF, LR, DT, and SVM in CNN. The ROC curve (receiver operating characteristic curve) of CNN, RF, LR, DT and SVM is shown in Fig. 2, and AUC (Area Under Curve) values are 1, 0.9689, 0.7807, 0.8009 and 0.7848 respectively, which also indicates that CNN is better than other methods.

We also use mouse and chicken data to compare the superiority of the convolutional neural network model (combined k-mers of *k* = 1, *k* = 2 and *k* = 3) in the classification of lncRNA sequences and mRNA sequences, and the results are shown in Table 6 (mouse) and Table 7 (chicken). CNN also has the highest model accuracy compared with the others. The model accuracy of the convolutional neural network algorithm is 0.8797 in mouse and 0.9963 in chicken.

**Table 6:** Five model effect comparison table in mouse

model	model accuracy	precision rate(P)	recall rate(R)	*F*_1_score
CNN	0.8797	0.8960	0.8590	0.8771
RF	0.8120	0.8132	0.8130	0.8131
LR	0.7541	0.7454	0.7700	0.7575
DT	0.7001	0.6991	0.6977	0.6984
SVM	0.7528	0.7564	0.7476	0.7520

**Table 7:** Five model effect comparison table in chicken

model	model accuracy	precision rate(P)	recall rate(R)	*F*_1_score
CNN	0.9963	0.9943	0.9984	0.9963
RF	0.9302	0.9351	0.9245	0.9298
LR	0.8743	0.8902	0.8546	0.8720
DT	0.8227	0.8148	0.8315	0.8230
SVM	0.8724	0.8881	0.8538	0.8706

### Verification of the classification model in single lncRNA sequence recognition

Our results are tested using 2000 mRNA sequences and 2000 lncRNA sequences selected from gencode.v 26 data. In addition, to verify whether the proposed classification model is suitable for the identification of a single sequence, we download a human lncRNA sequence in the NCBI database, which was discovered by Professor Gasri-Plotnitsky, an Israeli professor at the University of Barllan’s Institute of Life Sciences, and his team, and published in Oncotarget magazine in 2017 [25]. This lncRNA sequence is called GASL1. Professor Gasri-Plotnitsky’s research indicates that GASL1 expression inhibits cell cycle progression, identifying it as novel lncRNA modulator of cell cycle progression and cell proliferation, and has a potential role in cancer. Simultaneously, if the expression level of GASL1 is low in liver cancer patients, the survival rate may be worse.

Taking the GASL1 sequence as an example, we verify whether the classification model of the lncRNA and mRNA sequences proposed in this paper can correctly classify the sequences. The frequencies of 1-mers, 2-mers and 3-mers in the sequence are first calculated, and the three k-mers are combined together for a total of 84 k-mers. Finally, the frequencies of the 84 k-mers are constructed into a 7 × 12 matrix and convoluted. In the model constructed in this paper, the classification label of lncRNA is 0, and the classification label of mRNA is 1. To predict the category to which the sequence belongs, the output model is finally required to identify the category label of the category to which the sequence belongs.

The final prediction result is “pre_label is 0”, that is, the model recognizes the sequence as the lncRNA sequence, indicating that the model is correct for recognition of the sequence.

## Conclusions

The main purpose of this paper is to construct a model that can effectively classify lncRNA and mRNA. First, based on the statistical analysis of the sample sequence length and k-mer frequency distribution, the lncRNA and mRNA sequences in the model training set are determined to range from 250 nt to 3500 nt and from 200 nt to 4000 nt, respectively, and a k-mer frequency matrix is constructed. Then, using the k-mer frequency matrix as input in the convolutional neural network, a classification model of lncRNAs and mRNAs is established and programmed using Python. By calculating the classification accuracy of the frequency matrix of different k-mer combinations, the classification accuracy of the model with 1-mers, 2-mers and 3-mers is highest with an accuracy of 0.9872. Comparing the established lncRNA and mRNA classification models with random forest, logistic regression, decision tree and support vector machine analyses using the ROC curve, the model classification effect is improved. Application of the model is then examined: the correct classification result is obtained by identifying the known lncRNA sequence GASL1.

There remain many limitations to our research. For example, in the statistical analysis of the k-mers of lncRNA and mRNA sequences, only simple frequency analysis was used, and no in-depth statistical analysis was performed. In addition, when applying extensions to sequences of different species, the k-mer information difference between different species was not analysed in depth, but the preliminary discussion is based on the calculation results. In the future, we will conduct a systematic analysis of k-mer information differences between different species. In addition, as pointed out in [26] user-friendly and publicly accessible web-servers represent the future direction for developing practically more useful prediction methods and computational tools. Actually, many practically useful web-servers have significantly increased the impacts of bioinformatics on medical science [27], driving medicinal chemistry into an unprecedented revolution [28], we shall make efforts in our future work to provide a web-server for the prediction method presented in this paper.

## Methods

### Statistical analysis of k-mers of lncRNA and mRNA sequences

The human lncRNAs and mRNAs data were downloaded from the GENCODE database (Gencode.v26). The mouse lncRNAs and mRNAs data were downloaded from the GENCODE database (Genecode. VM21). The chicken lncRNAs and mRNAs data were downloaded from the Ensembl website database (5.0). Human data were used to build a convolutional neural network model. Mouse and chicken data were used to compare the superiority of the convolutional neural network model using the built model. The computed information used in this paper were as follows: (1) Operating system, Windows 10, with an InterCore I3–2365 M processor, memory size, 6G; (2) Python 3.5 to run the CNN code.

Studies have shown that the k-mer frequency information in lncRNA and mRNA sequences can reveal the distribution of various subsequences in biological sequences, to measure the similarities and variances of sequences [29].

**Fig. 3:**
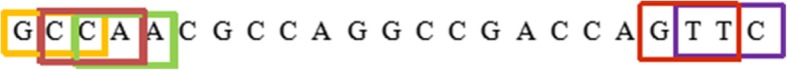
The 3-mer sliding window showing the process of taking a k-mer in sliding window mode in a sequence when k is three in which there are 21 3-mers

A k-mer refers to all possible subsequences of length k in a DNA sequence, RNA sequence or amino acid sequence. Figure 3 shows the process of examining a k-mer in a sliding window mode in a sequence when k is three, in which there are 21 3-mers, namely, GCC, CCA, CAA, AAC, ACG, CGC, GCC, CCA, CAG, AGG, GGC, GCC, CCG, CGA, GAC, ACC, CCA, CAG, AGT, GTT, and TTC. Among them, GCC and CCA appear three times, CAG appears twice, and the others appear once. Similarly, we can count the k-mer frequency information of lncRNA and mRNA sequences.

For a sequence, if the sequence length is m, then the number of k-mer subsequence of length k has m-k + 1. The sequence generally consists of four bases, A, T, C, and G, and thus k-mers of length k have 4^*k*^ possible structures.

**Fig. 4:**
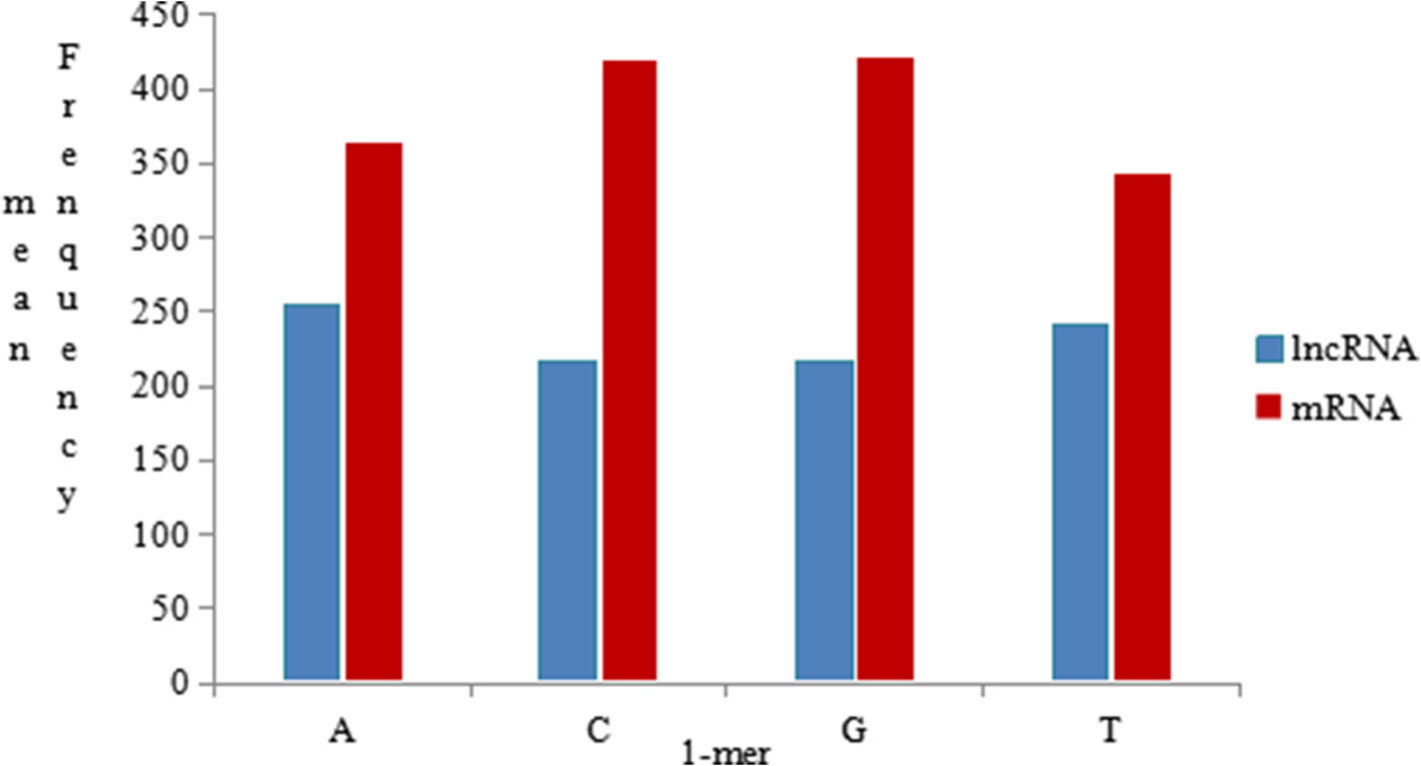
The 1-mer frequency distribution histogram. The contents of the A, C, G, and T bases in the lncRNA sequence are approximately 254 nt, 217 nt, 216 nt, and 240 nt, respectively, while the mRNA sequence has A, C, G, and T base contents of approximately 364 nt, 420 nt, 422 nt, and 343 nt, respectively, when we randomly select 5000 lncRNA sequence data and 5000 mRNA sequence data

**Fig. 5:**
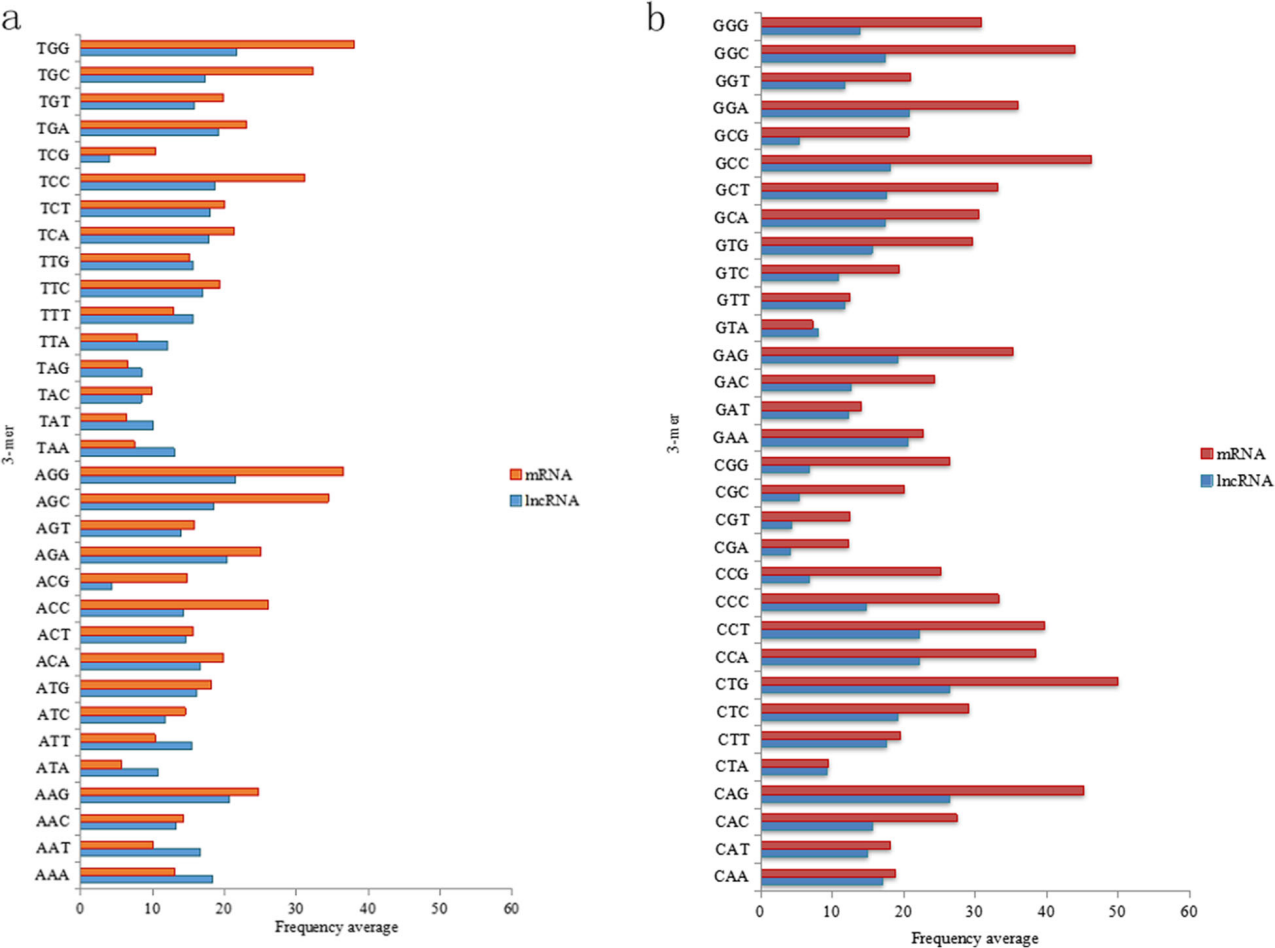
The 3-mer distribution frequency diagram of mRNA and lncRNA. **a** The 32 3-mer distribution frequency diagram beginning with T and A, and (**b**) the other 32 3-mer distribution frequency diagram beginning with G and C

We randomly selected 5000 lncRNA sequence data and 5000 mRNA sequence data from the human data, and we counted the k-mer frequency information when k = 1 and k = 3. As shown in Figs.4 and 5, when k = 1, the higher the content of bases G and C, the higher is the thermal stability of DNA molecules. When k = 2, after analysing the preference of dinucleotides, the frequency statistics of dinucleotides may represent certain characteristics of different species in different environments. For example, CG may be a methyl-CpG island and TA may be part of the TATA box. When k = 3, the coding and non-coding region in the sequence can be distinguished by counting the codon usage preference consisting of three bases. Therefore, considering the statistics, we analysed the k-mer frequency of lncRNA and mRNA sequence samples separately to discuss their differences.

From Fig. 4, the average contents of the A, C, G, and T bases in the lncRNA sequence were approximately 254 nt, 217 nt, 216 nt, and 240 nt, respectively, while the mRNA sequence had A, C, G, and T base contents of approximately 364 nt, 420 nt, 422 nt, and 343 nt, respectively. Therefore, the contents of the four 1-mers in the mRNA were higher than in the lncRNA. Furthermore, in the lncRNA and mRNA sequences, the contents of C and G bases were very similar, and the contents of A and T base were equivalent. However, in the lncRNA sequence, the contents of C and G base were lower than the contents of A and T base. While in the mRNA sequence, the opposite was true.

When k is taken as three, the 3-mer fragments appearing in the sequence are {AAA, AAT, AAC, AAG, ATA, ATT, ATC, ATG,..., GGA, GGT, GGC, GGG}. Similarly, the frequency information for each 3-mer segment of each sequence in the sample sequence is counted in turn, and the respective mean values are calculated to estimate the frequency of occurrence of each 3-mer in the lncRNA and mRNA sequences. The histogram of the 3-mer frequency distribution in the mRNA and lncRNA is plotted, as shown in Fig. 5a and b. As seen in Fig. 5a and b, the frequency distribution of most 3-mer subsequences in the mRNA sequence fluctuated sharply, but the frequency of 3-mers of GCG, CGG, CGC, CGT, CGA, TCG, and ACG was small, with only approximately 4 in each sequence. Moreover, the TGG, CAG, CTG, CCA, CCT, GCC, and GGC segments were enriched in the mRNA sequence, all with frequencies of approximately 40. In the lncRNA sequence, the frequency distribution of each 3-mer subsequence was relatively stable, and most of them were distributed approximately 15 with little fluctuation. A few 3-mers had a higher frequency, such as AAG, AGA, AGG, TGG, CAG, CTG, CCA, CCT, GAA, and GGA, exceeding 20. Moreover, only four 3-mers, ACG, TCG, CGA, and CGT, had frequencies lower than 5.

By analysing the frequency distributions of 1-mers and 3-mers of lncRNA and mRNA sequences, the k-mer distributions of the two were found to have their own preferences. Therefore, we could use the k-mer frequency distribution information for the sequence as the difference information between lncRNA and mRNA sequences.

### The lncRNA and mRNA classification model based on the convolutional neural network

**Fig. 6:**
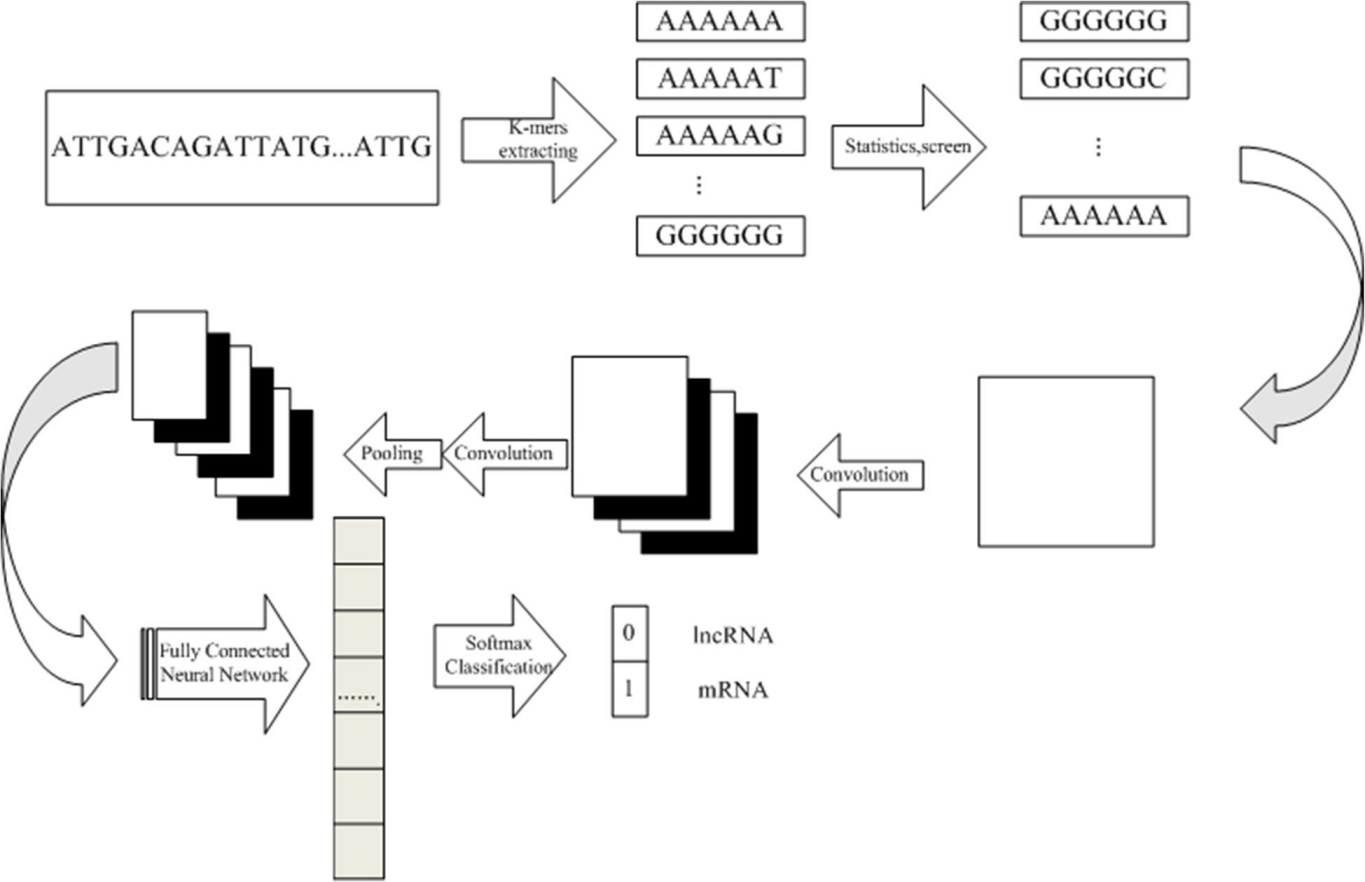
The lncRNA recognition model calculation flow chart. The lncRNA and mRNA classification model includes the input part and convolutional neural network part

In this paper, a convolutional neural network algorithm was used to construct a model suitable for classifying gene sequences based on the transformation of sequences into the k-mer frequency matrix. The model framework is shown in Fig. 6.

As shown in Fig. 6, the lncRNA and mRNA classification model includes the input part and the convolutional neural network part. The input section contains the k-mers extracted from the sequence and their construction into a k-mer frequency matrix. The convolutional neural network consists of two layers. First, in the k-mer feature extraction layer, the input of each neuron is connected to the local acceptance domain of the previous layer, and the local features are extracted. Once the local feature is extracted, its positional relationship with other k-mer features is also determined. The second is the k-mer feature mapping layer. Each computing layer of the network consists of multiple feature maps. Each feature map is a plane, and the weights of all neurons on the plane are equal. The k-mer feature mapping structure uses the sigmoid function as the activation function of the convolutional network so that the feature map has displacement invariance. In addition, since all neurons on a mapped surface share weights, the number of network parameters is reduced. Each convolutional layer in the convolutional neural network is followed by a computational layer for local averaging and quadratic extraction of k-mer features. This unique two-feature extraction structure reduces feature resolution.

### Construction of the k-mer frequency matrix

The k-mer frequency of each sequence is first normalized and converted to a frequency. Then, according to the application of the convolutional neural network in image recognition, the k-mer frequency of each sequence is constructed into a matrix form of the same size as the input of the model. Finally, the convolutional neural network is used to autonomously learn the difference between the two sequences of k-mer frequency information to achieve the purpose of classifying and identifying lncRNAs and mRNAs. The specific process is as follows:

- Step 1: The k-mer frequency information for each sequence is counted. In this paper, the sequence is traversed in the order of A, T, C, and G, and finally the frequency of each 4-mer is counted;
- Step 2: Normalize the frequency of each k-mer in the sequence in each sequence. That is, the frequency *p*_*i*_(*i* = *n*, *n* = 4^*k*^) of all k-mers in each sequence is obtained, and the sum of the frequencies of k-mers in each sequence is 1;
- Step 3: The frequencies of all k-mers in each sequence are constructed into a matrix form *A* × *B*(*A* × *B* = 4^*k*^), and the elements in the matrix are arranged horizontally in the order of the k-mer. For example, when k is 4, the constructed matrix is 16 × 16.

### K-mer screening based on relative entropy

When the value of k increases, the k-mer types with a length k in the sequence increase exponentially. If k is large, the average frequency of each k-mer will be less. Numerous k-mers have a frequency of zero. To reduce the complexity of the data calculation, we used relative entropy to screen k-mers.

Let *p*_ln*c*_ be the frequency distribution of the k-mer in the lncRNA sequence and *p*_*m*_ be the frequency distribution of the k-mer in the mRNA sequence. The relative entropy of *p*_ln*c*_ and *p*_*m*_ is then

1 $$ D\left({p}_{\ln c},{p}_m\right)={\sum}_{i=1}^{4^k}{p}_{\ln c}(i)\ln \frac{p_{\ln c}(i)}{p_m(i)},k\in \left[1,n\right],i\in \left[i,{4}^k\right], $$

If *p*_ln*c*_ = *p*_*m*_, then *D*(*p*_ln*c*_, *p*_*m*_) = 0, which indicates that the k-mer frequency distribution of the lncRNA sequence does not differ from the frequency distribution of the mRNA. If there is a difference in the k-mer frequency distribution between lncRNA and mRNA, then the value of *D*(*p*_ln*c*_, *p*_*m*_) will be greater than zero. Concurrently, the smaller the value of *D*(*p*_ln*c*_, *p*_*m*_) is, the smaller will be the difference in the k-mer frequency distribution between lncRNA and mRNA. Otherwise, the larger the value of *D*(*p*_ln*c*_, *p*_*m*_) is, the greater will be the difference in the k-mer frequency distribution between lncRNA and mRNA. To screen out k-mers that increase the difference information, set

2 $$ {d}_{\lambda }={p}_{\ln c}(i)\ln \frac{p_{\ln c}(i)}{p_m(i)},\lambda \in \left[i,{4}^k\right], $$

Sorting *d*_*λ*_ in descending order obtains

3 $$ R=\frac{\sum_{\lambda =1}^n{d}_{\lambda }}{D\left({p}_{\ln c},{p}_m\right)},n\in \left[1,{4}^k\right]. $$

*R* reflects the target ratio of the extracted information of k-mers. The *λ* corresponding to *R* from 1 to 4^*k*^ is sequentially calculated. If set *R* ≥ 98%, then calculate *λ* = *ϖ*, and the first *ϖ* k-mers are the filtered k-mers.

Specific steps are as follows:

- Step 1: The sum of each k-mer in the lncRNA sample sequence and the mRNA sample sequence is separately determined. Then, the frequency of each k-mer in it is counted. Finally, the frequency values of the 4^*k*^ kinds of k-mers in the sample are obtained. For example, when *k* = 4, the total frequency of 256 4-mers in the lncRNA and mRNA sample sequences are respectively counted, then the frequency value of each 4-mer is counted, and finally, 256 kinds of 4-mers are obtained. The sum of the frequency values of the 256 4-mers is 1 for the frequency values in the two sample sequences, respectively. The results of this step calculation are *p*_ln*c*_ and *p*_*m*_ in the formula (1).
- Step 2: According to the frequency value of each k-mer in the two sequences obtained in step 1, the relative entropy, that is, the value of *D*(*p*_ln*c*_, *p*_*m*_), is calculated according to the formula (1). Then, *R* is calculated according to the value of *D*(*p*_ln*c*_, *p*_*m*_) in formula (3), and finally, *λ* is taken as the value of *ϖ* when *R* ≥ 98%. Now according to the descending order of *d*_*λ*_, the first *ϖ* k-mers are the filtered k-mers.
- Step 3: The lncRNA and mRNA are separately counted based on the frequency of the k-mers screened in step 2, and then the k-mers frequency is constructed in the form of a matrix with reference to the steps of data input processing.

### Convolution calculation of the k-mer frequency matrix

The convolution calculation is used to strengthen the important features in the k-mer frequency matrix and weaken the influence of irrelevant k-mer features in this paper.

Taking *k* = 3 as an example, 64 k-mers can be extracted from an lncRNA sequence. According to the k-mer frequency, the lncRNA sequence can be constructed into an 8 × 8 k-mer frequency matrix *M*,

4 $$ M=\left[\begin{array}{cccccccc}0.0059& 0.0102& 0.0117& 0.0190& 0.0059& 0.0029& 0.0059& 0.0102\\ {}0.0088& 0.0073& 0.0220& 0.0059& 0.0220& 0.0146& 0.0234& 0.0146\\ {}0.0044& 0.0044& 0.0088& 0.0088& 0.0073& 0.0102& 0.0176& 0.0161\\ {}0.0132& 0.0176& 0.0322& 0.0117& 0.0117& 0.0220& 0.0249& 0.0146\\ {}0.0161& 0.0044& 0.0102& 0.0337& 0.0088& 0.0264& 0.0293& 0.0264\\ {}0.0278& 0.0439& 0.0366& 0.0190& 0.0044& 0.0132& 0.0190& 0.0102\\ {}0.0205& 0.0073& 0.0117& 0.0176& 0.0044& 0.0117& 0.0220& 0.0190\\ {}0.0161& 0.0220& 0.0366& 0.0102& 0.0146& 0.0073& 0.0176& 0.0161\end{array}\right]. $$

The convolution calculation is performed by taking Eq. (4) as input and randomly setting a convolution kernel of 3 × 3 ,

5 $$ \mathrm{Kernel}=\left[\begin{array}{ccc}1& 0& 1\\ {}0& 1& 0\\ {}1& 0& 0\end{array}\right]. $$

The convolution calculation is actually a process of weighted summation. The calculation format is usually

6 $$ {X}_j^l=f\left(\sum \limits_{i={M}_j}{X}_i^{l-1}\times {Kernel}_{ij}^l+{B}^l\right), $$

where *f* is the activation function of the neurons in the layer, and *l* indicates the number of layers in the network. The kernel is the convolution kernel, *M*_*j*_ is a local area of the input object, and *B* represents the offset of each layer.

In Eq. (5), the convolution kernel has nine parameters. The k-mer frequency matrix in Eq. (4) and the convolution kernel are convoluted by the calculation method of Eq. (6). *B*^*l*^ is set as 0. The specific calculation process is as shown in Eq. (7).

7 $$ {\displaystyle \begin{array}{c}\mathrm{feature}\ \mathrm{map}=\left[\begin{array}{cccccccc}{0.0059}_{\times 1}& {0.0102}_{\times 0}& {0.0117}_{\times 1}& 0.0190& 0.0059& 0.0029& 0.0059& 0.0102\\ {}{0.0088}_{\times 0}& {0.0073}_{\times 1}& {0.0220}_{\times 0}& 0.0059& 0.0220& 0.0146& 0.0234& 0.0146\\ {}{0.0044}_{\times 1}& {0.0044}_{\times 0}& {0.0088}_{\times 0}& 0.0088& 0.0073& 0.0102& 0.0176& 0.0161\\ {}0.0132& 0.0176& 0.0322& 0.0117& 0.0117& 0.0220& 0.0249& 0.0146\\ {}0.0161& 0.0044& 0.0102& 0.0337& 0.0088& 0.0264& 0.0293& 0.0264\\ {}0.0278& 0.0439& 0.0366& 0.0190& 0.0044& 0.0132& 0.0190& 0.0102\\ {}0.0205& 0.0073& 0.0117& 0.0176& 0.0044& 0.0117& 0.0220& 0.0190\\ {}0.0161& 0.0220& 0.0366& 0.0102& 0.0146& 0.0073& 0.0176& 0.0161\end{array}\right]\\ {}=\left[\begin{array}{cccccc}0.0293& 0.0556& 0.0323& 0.0527& 0.0337& 0.0467\\ {}0.0484& 0.0396& 0.0850& 0.0495& 0.0673& 0.0688\\ {}0.0469& 0.0498& 0.0480& 0.0644& 0.0657& 0.0776\\ {}0.0776& 0.0834& 0.1142& 0.0615& 0.0674& 0.0791\\ {}0.0907& 0.0820& 0.0497& 0.0821& 0.0557& 0.0835\\ {}0.0878& 0.0966& 0.0952& 0.0468& 0.0497& 0.0527\end{array}\right]\\ {}\kern2.75em \end{array}} $$

The first element of the feature map in Eq. (7) is the weighted sum of the first 3 × 3 local element value of the input matrix *M* and the corresponding element of the convolution kernel. Similarly, the second element is the weighted sum of the second 3 × 3 local element value and the corresponding element of the convolution kernel. Finally, a 6 × 6 size of the output matrix feature map is obtained.

The convolutional neural network of this model has two convolutional layers. The first convolutional layer uses 32 convolution kernels, and the second convolutional layer uses 64 convolution kernels. The size of each convolution kernel is 3 × 3, and the horizontal and vertical steps are 1. The border is filled with 0 in the samepadding to ensure that the size of the matrix remains the same as before convolution, i.e., the k-mer frequency matrix *M* after samepadding is

8 $$ {O}_j=\left[\begin{array}{cccccccc}0& 0& 0& 0& 0& 0& 0& 0\\ {}0& 0.0293& 0.0556& 0.0323& 0.0527& 0.0337& 0.0467& 0\\ {}0& 0.0484& 0.0396& 0.0850& 0.0495& 0.0673& 0.0688& 0\\ {}0& 0.0469& 0.0498& 0.0480& 0.0644& 0.0657& 0.0776& 0\\ {}0& 0.0776& 0.0834& 0.1142& 0.0615& 0.0674& 0.0791& 0\\ {}0& 0.0907& 0.0820& 0.0497& 0.0821& 0.0557& 0.0835& 0\\ {}0& 0.0878& 0.0966& 0.0952& 0.0468& 0.0497& 0.0527& 0\\ {}0& 0& 0& 0& 0& 0& 0& 0\end{array}\right]. $$

### Pooling calculation of the convolution kernel output matrix

The pooling method adopted in this paper is the max pooling, which aims to reduce and compress the k-mer characteristics, as well as the calculation amount. The model has only one pooling layer. After the second convolutional layer, the window sliding calculation is performed in a step size of 2.

For Eq. (8), the maximum value of the first 2 × 2 block of matrix *O*_*j*_ is 0.0293. The final result of the pooled calculation of matrix *O*_*j*_ is

9 $$ {S}_j=\left[\begin{array}{cccc}0.0293& 0.0556& 0.0527& 0.0467\\ {}0.0484& 0.0850& 0.0673& 0.0776\\ {}0.0907& 0.1142& 0.0821& 0.0835\\ {}0.0878& 0.0966& 0.0497& 0.0527\end{array}\right]. $$

Since 64 k-mer matrices of 6 × 6 were obtained after the second convolutional layer and the size of the pooling window was 2 × 2, the number of rows and columns of the matrix became half the original, representing 64 k-mer 3 × 3 matrices.

### Fully connected neural network based on SoftMax function

In order to prevent over-fitting and improve the generalization ability of the model, we reduce the connection of some neurons with a certain probability, so that some neurons in each training are not activated. After the pooling layer, the connection between the output neurons of the pooling layer and the neurons of the full connective layer was reduced with a probability of 0.25. The output matrix of the pooling layer is flattened and expanded to connect 128 neurons in the full connection layer. The activation function is still Relu function.

We used the SoftMax function to activate the output of the fully connected network in the model. The formula of the SoftMax function is

10 $$ f\left({z}_j\right)=\frac{e^{z_j}}{\sum_{i=1}^n{e}^{z_i}}. $$

From Eq. (10), if *z*_*j*_ is greater than the other *z*, the value of the function *f*(*z*_*j*_) approaches 1, and otherwise it approaches 0. Therefore, when the value of *f*(*z*_*j*_) is 1, the input sequence of this model is judged to be an lncRNA sequence, and when the value of *f*(*z*_*j*_) is 0, the input sequence is a mRNA sequence.

The Adadelta optimizer is used to train the gradient descent in the training process, and the cross-entropy loss function is used as the loss function.

### Setting of the evaluation index in the classification model

The indicator for evaluating the performance of a classification model is generally the classification accuracy, which is also known as the model accuracy. Commonly used evaluation indicators for the two-category problem are precision and recall. In this paper, the positive class was the lncRNA sequence, and the mRNA sequence was the negative class. The predictions of the classifier on the test data set were either correct or incorrect. The total number of occurrences in the four cases was recorded as follows:

*TP*——The positive class is predicted as the positive class number;

*FN*——The positive class is predicted as the negative class number;

*FP*——The negative class is predicted as the positive class number;

*TN*——The negative class is predicted as the negative class number.

The precision rate is defined as

11 $$ P=\frac{TP}{TP+ FP}, $$

The recall rate is defined as

12 $$ R=\frac{TP}{TP+ FN}. $$

In addition, the *F*_1_ score is the harmonic mean of the precision rate and the recall rate, i.e.,

13 $$ \frac{2}{F_1}=\frac{1}{P}+\frac{1}{R}, $$

14 $$ {F}_1=\frac{2 TP}{2 TP+ FP+ FN}, $$

If both the precision and recall rates are high, then *F*_1_ will be high [30].

## Data Availability

The datasets used to perform the analysis are publicly available at https://www.gencodegenes.org/mouse/

## Abbreviations

ANT: Adjoining Nucleotide Triplets
AUC: Area Under Curve
CNCI: Coding-Non Coding Index
CPC: Coding Potential Calculator
DT: Decision Tree
lncRNA: Long-chain non-coding RNA
LR: Logistic regression
MLCDS: Most-Like Coding Domain Sequence
mRNA: Messenger RNA
RF: Random Forest
ROC curve: Receiver operating characteristic curve
SVM: Support Vector Machine
